# **Populism and Health Policy in Latin America** Comment on "A Scoping Review of Populist Radical Right Parties’ Influence on Welfare Policy and its Implications for Population Health in Europe"

**DOI:** 10.34172/ijhpm.2020.153

**Published:** 2020-08-11

**Authors:** Mary A. Clark, Amy Patterson

**Affiliations:** ^1^Department of Political Science, Tulane University, New Orleans, LA, USA.; ^2^Department of Politics, University of the South, Sewanee, TN, USA.

**Keywords:** Populism, Latin America, Health Policy

## Abstract

This commentary focuses on Latin America, a region known for its rich variety of populist politicians and some of the most extensive welfare states in the Global South. Contemporary Latin America offers examples of left-wing and right-wing populist leaders, none of whom demonstrate the same focus on excluding immigrants from welfare state benefits as that noted by Chiari Rinaldi and Marleen Bekker in the European context. We see this contrast not because immigrants’ access to health services is less important in Latin America, but because Latin American populists are more focused on internal "enemies." The commentary concludes with observations regarding Latin American populist leaders’ handling of the Coronavirus disease 2019 (COVID-19) pandemic.

In “A Scoping Review of Populist Radical Right Parties’ Influence on Welfare Policy and its Implications for Population Health in Europe,” Chiari Rinaldi and Marleen Bekker review the literature on populist radical right parties and welfare state policy in Europe.^1^ They are interested in how an ideology known as “welfare chauvinism,” a common priority among such European parties, might affect population health. This ideology expresses an aspect of majoritarian identity politics that attempts to remove welfare state benefits from those who are perceived as not belonging in the country, ie, immigrants. While in several present-day European countries there is a causal connection between the rise of right-wing populism, welfare state nativism, and health policy decisions, we need to ask how other world regions compare. Here we focus on Latin America, a region known for its rich variety of populist politicians and some of the most extensive welfare states in the Global South. Contemporary Latin America offers examples of left-wing and right-wing populist leaders whose programs reflect different concerns about health policy.

Populism is a notoriously flexible concept and observers have acknowledged the ideological and organizational diversity among such leaders. Ideologically, populism is anchored by the notion that corrupt elites are the antithesis of “the people,” but is otherwise thin and malleable.^2^ As a political strategy, populism relies on a personalistic leader who mobilizes followers in a variety of ways ranging from party machines to direct appeals through mass media outlets.^3^ Thus, populism is a view of the polity and a strategy for gaining power that can be used by leaders on the left, right, and anywhere in between. Such leaders share a common claim of fighting enemies to deliver justice to the populace and a refusal to be hemmed in by institutional checks and balances or party procedures.^4^

The inclusiveness of traditional Latin American populism has long contrasted with the typically exclusionary European variety.^2^Historically, populist leaders such Juan Perón of Argentina harnessed the voting power of previously excluded urban workers. In exchange for their electoral support, such leaders strengthened welfare state benefits for the working class including union-based health funds.^5^ Such governments often endorsed economic policies that protected nascent domestic manufacturing firms from international competition. Populist leaders such as Perón wrapped up these policies in rhetoric emphasizing the just redistribution of economic benefits within society and defense against the economic imperialism of wealthier countries. Several recent populist leaders have resurrected anti-imperialist and redistributive rhetoric including Hugo Chávez in Venezuela (1999-2013), Evo Morales in Bolivia (2006-2019), Cristina Kirchner in Argentina (2007-2015), and Rafael Correa in Ecuador (2007-2017). The current president of Mexico, Andrés Manuel López Obrador (widely known as AMLO), is a less radical left-of-center populist leader. More unusual in Latin America are two recently elected right-wing populist presidents, Jair Bolsonaro of Brazil and Nayib Bukele of El Salvador. Bolsonaro exemplifies a new form of exclusionary populism; he shares with the milder Bukele a predilection for burnishing his law-and-order credentials with highly visible deployments of security forces.

Unlike the European context, the inclusion or exclusion of immigrant populations is not a large part of the public appeal for the current crop of populist leaders in Latin America. Rather, the main target of Latin America’s populist leaders of all stripes is the political establishment. Established political parties and their leaders are seen as corrupt and also inept at solving pressing problems such as violent crime. Many Latin Americans feel that corrupt politicians and violent criminals benefit from equal impunity.^6^ In Brazil, Bolsonaro’s election came after several years of economic recession, rising violent crime rates, and corruption scandals involving the former and sitting presidents of the programmatic, leftist Workers’ Party. Along with capitalizing on anger at politicians, Bolsonaro allied himself with conservative segments of society bothered by affirmative action measures installed by previous progressive administrations on behalf of Afro-Brazilians and protections granted to the LBGTQ community and indigenous Amazonian tribes. Like Bukele in El Salvador, Bolsonaro also seeks to effectively exclude suspected violent criminals and gang members from equal protection under the law. Even for the right-wing Latin American populists, then, the “outgroups” they attack are domestic citizens.

None of this is to say that the issues of immigration, legal citizenship, and access to welfare-state benefits, including health, are not real in Latin America, only that they are not, as yet, mixed up in populist politics as in the European examples highlighted by Rinaldi and Bekker. For example, over the last several decades Costa Rica has wrestled with how to handle the heavy use of its public healthcare system by noncontributory immigrants, almost entirely from Nicaragua, who compose about 10 percent of the country’s population.^7^ The solution has been to formalize their status and require both the immigrants and their employers to pay quotas into the public health insurance system. Other countries have opted for exclusionary policies by refusing legal recognition of immigrants and citizenship to their descendants, thus denying them access to public health insurance programs. This is a long-standing problem in the Dominican Republic where generations of people of Haitian descent have been denied citizenship.^8^ Mexico’s stance toward Central American immigrants is somewhere in the middle. Its public health program for the uninsured covers undocumented people for 90 days but immigration laws make it quite difficult for migrants to obtain the residency status required for long-term enrollment.^9,10^ None of these policies were prioritized by populist leaders but that is always a possibility, particularly in the Andean nations whose health systems are being challenged by the needs of displaced Venezuelans.^11,12^

Several recent left-wing populist administrations in Latin America have demonstrated inclusiveness by posting more doctors to work in poor areas of their countries. Instead of reforming existing institutions, these are add-on programs. For example, as president of Venezuela, Hugo Chávez devised to bring medical services to the poorest by sending oil to Cuba in exchange for physicians from that country. Shortly after the turn of the century, Venezuela was hosting 19000 Cuban doctors.^13^ They were part of Chávez’ *Barrio Adentro* (Inside the Neighborhood) program, assigned to live in the poorest areas of the country and offer free services to residents of those neighborhoods.^14^Presidents Morales in Bolivia and Correa in Ecuador pursued smaller programs with Cuban doctors. Non-populist governments also contracted with the Cuban government to send physicians to remote postings and impoverished communities. For instance, the Brazilian government of Dilma Rousseff, representing the Workers’ Party, launched the *Mais Médicos* (More Doctors) program in 2013. By the following year, more than 11000 Cuban doctors were working in the country.^13^*Mais Médicos* did attract the attention of President Bolsonaro, however, who cancelled the program as soon as he took office.

Have populist presidents reshaped existing health systems to serve the goals of social inclusion or exclusion? Mexico’s “system” remains segmented between a small private insurance market, social security schemes, and a national program called, until recently, *Seguro Popular *(People’s Health Insurance). *Seguro Popular *was aimed at the poorest members of society, many of whom worked in the informal sector. AMLO reformulated *Seguro Popular *into a new institution, *Instituto de Salud para el Bienestar* (Institute of Health for Welfare), whose services and medications are free, something that was not always the case under the old program. As a left-of-center populist, AMLO has behaved as we would expect by making good on a campaign pledge to extend free healthcare to the poor and associating the new program with himself.

In Brazil, President Bolsonaro has been disinterested in healthcare and more concerned with reforming the pension system and federal civil service statutes.^15^ The right to universal healthcare was written into the 1988 constitution that accompanied the return to democracy after decades of military dictatorship. Rather than a mere aspiration, the constitutional guarantee to coverage by the Unified Health System (*Sistema Único de Saúde*) is strongly valued by Brazilians despite the system’s imperfections.^16^ In any case, Bolsonaro does not need to propose new measures to discipline public health spending because a draconian austerity law was already in place before he took office in 2019. In 2015, Brazil plunged into a deep economic recession and in 2016, convinced that runaway public-sector spending was driving the problem, the national congress passed Constitutional Amendment 95, which set a ceiling on government spending for 20 years. Total government spending cannot increase beyond the previous year’s amount plus inflation. Given population growth, we can expect per capita public health expenditures to fall over time.

The coronavirus disease 2019 (COVID-19) crisis provides a window onto how populist presidents respond to health emergencies. One notable aspect of populist leaders’ strategy of representing ordinary people is that they sometimes exhibit an anti-intellectualism that encompasses resistance to expert knowledge.^17^ Regardless of their ideological orientation on the left-right scale, such leaders may frame scientific agencies, including global allies such as the World Health Organization (WHO), as one more type of elite establishment with whom they are battling for control. For example, Presidents Bolsonaro in Brazil and AMLO in Mexico have little in common beyond the obstinate denial of the seriousness of the COVID-19 pandemic for their citizenry. They have rejected advice from their ministers of health and medical organizations, refused to wear masks, mingled with citizens in the streets, and encouraged supporters to hold rallies.^18,19^Alternatively, a populist leader may declare a state of emergency that grants him extra-constitutional powers. In El Salvador, President Bukele responded to the pandemic with a draconian state of emergency and defied decisions by the legislative assembly and constitutional court to end it or to allow oversight of associated public spending.^20^ Bukele has also deployed troops to crackdown on gangs with lethal force during the COVID-19 quarantine.^21^

 In conclusion, we do not see the same connections between populism and welfare chauvinism in Latin America. This is not because the question of immigrant access to welfare state health benefits is irrelevant in Latin America nor because right-wing populism is unknown in the region. The picture of Latin American populism as inclusive remains generally true, although there are important exceptions, President Bolsonaro of Brazil being the most glaring of these. Had Amendment 95 not already been in place, Bolsonaro may have been tempted to make drastic cuts in public health spending. But he would risk strong political disapproval for pulling back on the universal nature of the constitutionally enshrined Unified Health System. To be sure, in-depth research on these links might well complicate the conclusions drawn in this brief commentary.

 On a different note, the COVID-19 crisis offers the opportunity to witness how Latin American populists handle an enormous health emergency. Here the populist leader’s need to maintain personal control over the state apparatus is evident but manifested in different ways. Several populist leaders have responded by refusing to submit to the authority of the scientific establishment whose knowledge they deride as elitist discourse. Nayib Bukele in El Salvador pursued a different strategy by accepting medical advice and using a stringent national lockdown to grab more power for himself.

## Ethical issues

 Not applicable.

## Competing interests

 Authors declare that they have no competing interests.

## Authors’ contributions

 Both contributed to the equally to the conceptualization, writing, and editing of this manuscript.

## Authors’ affiliations

^1^Department of Political Science, Tulane University, New Orleans, LA, USA. ^2^Department of Politics, University of the South, Sewanee, TN, USA
